# TSGΔ154-1054 splice variant increases TSG101 oncogenicity by inhibiting its E3-ligase-mediated proteasomal degradation

**DOI:** 10.18632/oncotarget.6973

**Published:** 2016-01-22

**Authors:** Huey-Huey Chua, Chiun-Sheng Huang, Pei-Lun Weng, Te-Huei Yeh

**Affiliations:** ^1^ Graduate Institute of Microbiology, College of Medicine, National Taiwan University, Taipei 10051, Taiwan; ^2^ Department of Surgery, National Taiwan University Hospital, College of Medicine, National Taiwan University, Taipei 10051, Taiwan; ^3^ Department of Otolaryngology, National Taiwan University Hospital, College of Medicine, National Taiwan University, Taipei 10051, Taiwan

**Keywords:** TSG101, alternative splicing, ubiquitination, tumorigenesis, nasopharyngeal carcinoma

## Abstract

Tumor susceptibility gene 101 (TSG101) elicits an array of cellular functions, including promoting cytokinesis, cell cycle progression and proliferation, as well as facilitating endosomal trafficking and viral budding. TSG101 protein is highly and aberrantly expressed in various human cancers. Specifically, a TSG101 splicing variant missing nucleotides 154 to 1054 (TSGΔ154-1054), which is linked to progressive tumor-stage and metastasis, has puzzled investigators for more than a decade. TSG101-associated E3 ligase (Tal)- and MDM2-mediated proteasomal degradation are the two major routes for posttranslational regulation of the total amount of TSG101. We reveal that overabundance of TSG101 results from TSGΔ154-1054 stabilizing the TSG101 protein by competitively binding to Tal, but not MDM2, thereby perturbing the Tal interaction with TSG101 and impeding subsequent polyubiquitination and proteasomal degradation of TSG101. TSGΔ154-1054 therefore specifically enhances TSG101-stimulated cell proliferation, clonogenicity, and tumor growth in nude mice. This finding shows the functional significance of TSGΔ154-1054 in preventing the ubiquitin-proteasome proteolysis of TSG101, which increases tumor malignancy and hints at its potential as a therapeutic target in cancer treatment.

## INTRODUCTION

Tumor susceptibility gene 101 (TSG101) encodes a cellular endosomal sorting complex protein required for transport (ESCRT)-I protein, which form a multi-complex vesicular transport machinery [1]. This machinery controls the sorting of ubiquitinated proteins to the endosomes, facilitating receptor traffic and turnover, and has been implicated in normal development, cell differentiation, and growth, as well as the budding of certain enveloped viruses. Dysregulation of ESCRT proteins has been correlated with human diseases, including many types of cancers and neurodegenerative disorders [2]. Although *TSG101* was originally discovered in a screen for potential tumor suppressor genes [3], it is a potential oncogene with multiple cellular functions. TSG101 was found to be crucial for embryonic tissue survival and proliferation using *TSG101* knockout mice [4, 5]. Also, TSG101 deficiency causes cell cycle arrest at G1/S transition in primary embryonic fibroblasts and tumor cell lines in cell culture systems [6], and TSG101 depletion leads to reduced tumor cell clonogenicity, migration, and drug resistance [7, 8]. Additionally, TSG101 serves as a transcriptional co-regulator in suppressing transcription of nuclear hormone receptors and p21 (CIP1/WAF1) tumor suppressor gene [9–11].

During the progression of tumorigenesis of a variety of human cancers, a stage-dependent deregulation of alternative splicing programs frequently occurs, correlating to the acquisition of malignant features including vigorous proliferation, anti-apoptotic capabilities, metastasis, invasion, and poor prognosis [12, 13]. Li et al. discovered the presence of alternatively spliced *TSG101* transcripts in breast cancers using reverse transcription (RT)-nested PCR [14]. Numerous reports agree on the increased frequency of TSG101 splicing abnormalities in cervical, lung, prostate, and other cancers [15–18]. The TSG101 splice variant, which harbors a deletion of nucleotides 154 to 1054 (designated TSGΔ154-1054), is the most commonly expressed in human tumor tissues [14–18]. Increased TSGΔ154-1054 transcription in pre-neoplastic lesions, as well as malignant biopsies of cervical cancer, correlate with neoplastic progression [15]. Additionally, the TSGΔ154-1054 transcript is frequently present in late-stage breast cancer and correlates with advanced axillary lymph node metastasis [19]. We have also demonstrated that TSG101 cooperated with a viral lytic transactivator Rta to promote late genes activation of Epstein-Barr Virus (EBV), a DNA virus related with the development of nasopharyngeal carcinoma (NPC) [20]. In follow-up studies, we found a direct correlation between TSGΔ154-1054 expression and abundance of TSG101 protein in both NPC and breast cancer. These findings evoked our interest to investigate the detailed relationship between the TSG101 protein and its TSGΔ154-1054 splice variant.

In mammalian cells, the amount of TSG101 protein is strictly balanced via a post-translational process involving its ubiquitin (Ub)-proteasome proteolysis [21, 22]. A conserved RING E3 Ub ligase, TSG101-assaciated ligase (Tal), negatively regulates TSG101 abundance by targeting TSG101 for ubiquitination with subsequent degradation [21, 23]. MDM2, another E3 Ub ligase, also accelerates the turnover of excess TSG101 and takes part with TSG101 in a regulatory loop connecting the MDM2-p53 circuit [22]. Furthermore, the post-translational autoregulation of TSG101 controls the total intracellular pool of TSG101 protein at a steady-state level [24]. Although there are several alternate degradative routes governing the amount of TSG101, increased TSG101 protein is still frequently shown in a variety of cancers [25–27].

In the light of high incidence of increased TSG101 protein found together with its splice variant in NPC and breast tumor progression, we report herein that the expression of TSGΔ154-1054 prevents the polyubiquitination and degradation of TSG101 protein by binding competitively to Tal, and subsequently increased the oncogenicity of TSG101 in terms of enhancing cell proliferation and malignant tumor formation in nude mice.

## RESULTS

### Comparison of full-length (FL)-TSG101 and TSGΔ154-1054

**Figure 1:**
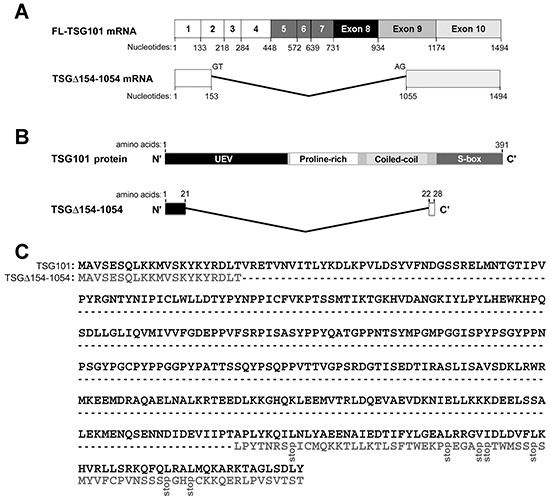
TSGΔ154-1054 mRNA and protein **A.** Structures of full-length (FL)-TSG101 and TSGΔ154-1054 mRNA. Numbered boxes illustrate the coding exons. **B.** Schematic illustration of the domain structures of TSG101 and TSGΔ154-1054. **C.** Alignment of the TSG101 amino acid sequence and the presumptive sequence of TSGΔ154-1054. A dashed line represents the amino acids deleted from the TSGΔ154-1054, compared to TSG101.

The genomic structure, functional units, and amino acid sequence of the TSG101 gene and deduced sequence of the TSGΔ154-1054 variant are shown in Figure 1A and 1B. Human TSG101 protein is 391 amino acids long and contains four known structural domains: the N-terminal ubiquitin E2 variant (UEV) binding domain, followed by a proline-rich region (PRR), a coiled coil (CC) region, and a C-terminal α-helical/steadiness box (S-box) domain (Figure 1B). Figure 1C demonstrates the alignment of the TSG101 amino acid sequence (NCBI accession number U82130) and the presumptive sequence of TSGΔ154-1054. A dashed line denotes the amino acids deleted in TSGΔ154-1054, compared to TSG101. The presumptive TSGΔ154-1054 protein product is composed of 28 amino acids with a molecular weight of nearly 3 kDa. The N-terminus of the TSGΔ154-1054 protein is identical to the N-terminus of full length TSG101. The TSGΔ154-1054 C-terminus differs completely from the coordinate sequences of full length TSG101 because of a frameshift, and terminates prematurely before the S-box, which is responsible for autoregulation of the steady state level of TSG101 (Figure 1C) [24]. Endogenous TSGΔ154-1054 protein product detection is shown in Supplementary Figure S1. Obviously, posttranslational modification exists for this small protein.

### TSGΔ154-1054-mediated stabilization of TSG101 protein

**Figure 2:**
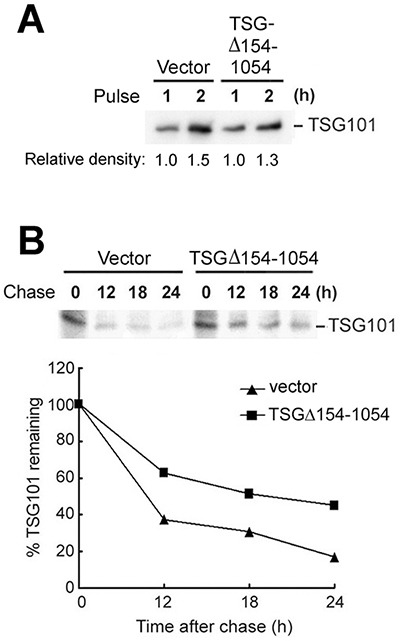
TSGΔ154-1054 perturbs the degradation of TSG101 protein **A-B.** TW01 cells were transfected with control vector or TSGΔ154-1054 expression plasmids; then, the transfectants were pulsed (A) in ^35^S-[Met]-containing medium and chased (B) for the times indicated. Following immunoprecipitation with anti-TSG101 antibody, the immunocomplexes were analyzed by autoradiography (upper panel). One representative result of three independent experiments is shown. The degradation of endogenous TSG101 is plotted in the lower panel.

To investigate the possibility that TSGΔ154-1054 exists without redundancy but positively controls the total pool of TSG101, ^35^S pulse-labeling was performed to detect newly synthesized protein. TSGΔ154-1054 expression did not change the amount of TSG101 produced (Figure 2A). On the other hand, in a kinetic assay for TSG101 turnover, TSGΔ154-1054 expression extended the half-life of TSG101 (Figure 2B), indicating that TSG101 protein is stabilized by TSGΔ154-1054. This finding was verified in NPC tissues and in two other NPC cell lines, HONE1 and TW04; the levels of TSG101 proteins were concomitantly elevated upon expression of endogenous and exogenous TSGΔ154-1054 in NPC tissues and cell lines, respectively (Supplementary Figure S1C and S2). Increased TSG101 was noted in the protein but not the transcriptional level (Supplementary Figure S2), supporting the finding that TSG101 protein is stabilized by TSGΔ154-1054.

### TSGΔ154-1054 protects TSG101 from proteasomal degradation mediated by Tal

**Figure 3:**
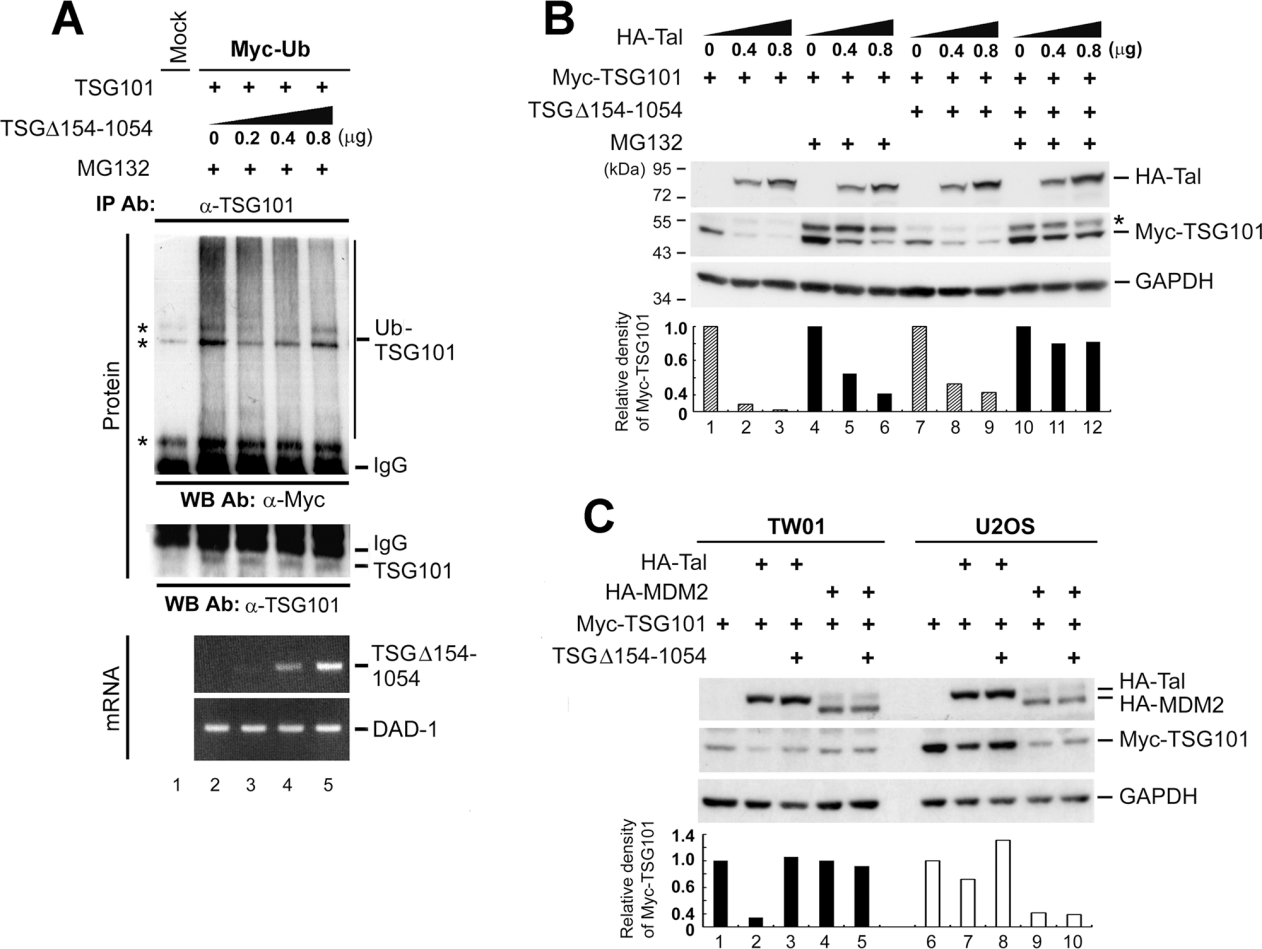
TSGΔ154-1054 protects TSG101 from proteasomal degradation mediated by Tal but not MDM2 **A.** TW01 cells were co-transfected with the respective expression plasmids. MG132 (10 mM) treatment was administered at 18 h post-transfection and incubated for 6 h before harvesting. Immunoprecipitation-western blot assay was carried out to assess the amounts of Myc-Ub -polymerized TSG101. IgG specifies the heavy chain of the immunoglobulin G. Asterisks indicate non-specific cross-reactive bands. RT-PCR analysis (Bottom) was conducted to demonstrate the expression levels of transfected TSGΔ154-1054 transcripts and DAD-1 served as cDNA loading control. **B.** Effect of TSGΔ154-1054 on Tal-mediated TSG101 proteolysis. TW01 cells introduced with HA-Tal as indicated, Myc-TSG101 (1 μg), and TSGΔ154-1054 (0.4 μg) plasmids were administrated with MG132 as described above, and harvested for western blot analysis at 24 h post-transfection. Asterisk indicates an unidentified cross-reactive band. **C.** Comparison of the effects of TSGΔ154-1054 on Tal- and MDM2-mediated TSG101 degradation. Both TW01 and U2OS cells were transfected with the plasmids indicated and harvested at 24 h for western blot analysis. Densitometric analysis of Myc-TSG101 was accomplished by means of IMAGEQUANT. Similar results were obtained in three independent experiments.

TSG101 is eliminated through the Ub-mediated proteasomal degradation pathway [22, 25]. If TSGΔ154-1054 represses the turnover of TSG101, then the inhibition of TSG101 polyubiquitination is expected to occur in the presence of TSGΔ154-1054. A wild-type Ub construct (Myc-Ub) was used to test this effect. Co-expression of Myc-Ub and TSG101 in TW01 cells resulting in a pattern of heavily polyubiquitinated TSG101 (Figure 3A, lane 2). On the contrary, increasing expression of TSGΔ154-1054 reduced the level of TSG101 polyubiquitination (Figure 3A, lanes 3-5), suggesting that TSGΔ154-1054 prevents the polyubiquitination of TSG101 thereby rescuing it from degradation.

Because Tal ubiquitinates and targets TSG101 protein for degradation [23, 25], we postulated that the protective role of TSGΔ154-1054 in TSG101 degradation may involve obstructing the action of Tal. In Figure 3B, expression of HA-Tal drove Myc-TSG101 degradation (lanes 1-3), while co-expression of TSGΔ154-1054 prevented the direct degradative effect of Tal on Myc-TSG101 (lanes 7-9). Treatment with MG132, a proteasome inhibitor, rescued the majority of Myc-TSG101 from proteolysis (Figure 3B, lanes 4-6), and the protection of TSG101 by TSGΔ154-1054 became more evident (Figure 3B, lanes 10-12), implying that TSGΔ154-1054 indeed interrupts Tal-mediated proteasomal degradation of TSG101.

Considering that the E3 ligase activities of both Tal and MDM2 control the Ub-dependent proteolysis of TSG101 [22], we tested the effect of TSGΔ154-1054 on MDM2-mediated TSG101 degradation. In TW01 cells, which express a normal level of MDM2 [28], TSGΔ154-1054 prevented only HA-Tal, but not HA-MDM2, mediated Myc-TSG101 degradation (Figure 3C). In U2OS, which harbor a single copy of the *MDM2* gene and express a negligible amount of MDM2 [22], the level of Myc-TSG101 protein diminished obviously when HA-MDM2 was overexpressed; however, the presence of TSGΔ154-1054 did not rescue Myc-TSG101 from HA-MDM2-mediated degradation (Figure 3C, lane 10). Thus, TSGΔ154-1054 acts only by disturbing Tal-mediated proteolysis to stabilize TSG101.

### TSGΔ154-1054 competes with TSG101 for binding to Tal, a requisite for diminishing Tal-mediated polyubiquitination of TSG101

**Figure 4:**
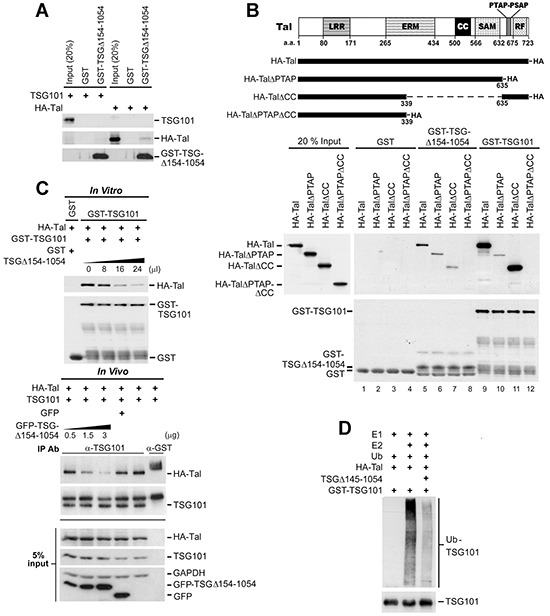
TSGΔ154-1054 competes with TSG101 for binding Tal thereby diminishing Tal-mediated polyubiquitination of TSG101 **A.** Binding of TSGΔ154-1054 to Tal but not TSG101. Glutathione-Sepharose-bound GST-TSGΔ154-1054 and the *in vitro* translated HA-Tal or TSG101 were applied to *in vitro* binding assay followed by western blotting. **B.** Binding of Tal and its deletion mutants to TSGΔ154-1054 and TSG101. A schematic illustration of the domain structures of Tal and its deletion mutants is shown (top). Tal comprises a leucine-rich repeat (LRR), ezrin-radixin-moesin (ERM) domain, coiled-coil (CC) region, sterile alpha motif (SAM), RING finger (RF), and a double PTAP motif. These HA-Tal mutants were subjected to *in vitro* transcription and translation, and applied to *in vitro* binding assay together with bead-bound GST-TSGΔ154-1054 or GST-TSG101. The result was visualized by western blotting (bottom). **C.** Competitive binding between TSGΔ154-1054 and TSG101 to Tal. Glutathione-Sepharose-bound GST-TSG101 and the *in vitro* translated HA-Tal were subjected to an *in vitro* competitive binding assay in the presence of increasing amounts of *in vitro* translated TSGΔ154-1054, and the result was revealed by western blotting (upper panel). An *in vivo* competitive binding between TSGΔ154-1054 and TSG101 to Tal was accomplished on TW01 cells co-transfected with HA-Tal, TSG101 and increasing level of GFP-TSGΔ154-1054 using co-immunoprecipitation coupled with western blot (lower panel). Co-immunoprecipitation was performed using anti-TSG101 antibody, and anti-GST antibody as an irrelevant antibody control. **D.** Effect of TSGΔ154-1054 on Tal-mediated ubiquitination of TSG101. The bead-bound GST-TSG101 was incubated with an i*n vitro* ubiquitination reaction containing E1, E2 (UbcH5a), Ub, immunopurified HA-Tal, and *in vitro* translated TSGΔ154-1054. Immunoblot was performed using anti-Ub antibody.

In view of Tal degrading TSG101 through a direct interaction [23, 25], we reasoned that the restraining effect of TSGΔ154-1054 on the degradative activity of Tal may result from impeding the substrate recognition of Tal. An *in vitro* binding assay was performed to elucidate the probability of TSGΔ154-1054 binding directly to TSG101 or Tal. As shown in Figure 4A, GST-TSGΔ154-1054 interacts with Tal, but not TSG101. A series of Tal deletion mutants was cloned subsequently and an *in vitro* binding assay for Tal mutants and GST-TSGΔ154-1054 was performed in comparison with that of GST-TSG101 (Figure 4B). The result resembled a previous report that shows the Pro–Thr–Ala–Pro (PTAP) motif of Tal is the main determinant of TSG101 binding while the CC domain displayed weaker binding to GST-TSG101 [25]; whereas both the PTAP motif and CC domain of Tal contributed equivalently to interact with GST-TSGΔ154-1054 (Figure 4B). If TSGΔ154-1054 binds Tal, we postulated that TSGΔ154-1054 may compete with TSG101 for binding Tal. *In vitro* and *in vivo* competitive binding assays consistently illustrated a greater amount of TSGΔ154-1054, and a lesser amount of HA-Tal, bound to TSG101 (Figure 4C). It seems that, when they co-exist, TSGΔ154-1054 and TSG101 compete to bind Tal.

To determine the consequences to TSG101 when TSGΔ154-1054 binds to Tal, we investigated the cognate E2 Ub-conjugating enzyme for Tal ubiquitination of TSG101 and found that it was ubcH5a (data not shown). In a subsequent *in vitro* ubiquitination assay, we observed that the E3 ligase activity of Tal for GST-TSG101 ubiquitination was substantially reduced in the presence of TSGΔ154-1054 (Figure 4D). This hints to a consequential reduction in Ub-dependent degradation of TSG101 when TSGΔ154-1054 competes with TSG101 to bind to Tal.

### TSGΔ154-1054 functionally augments the biological activities of TSG101 by increasing TSG101 stability

**Figure 5:**
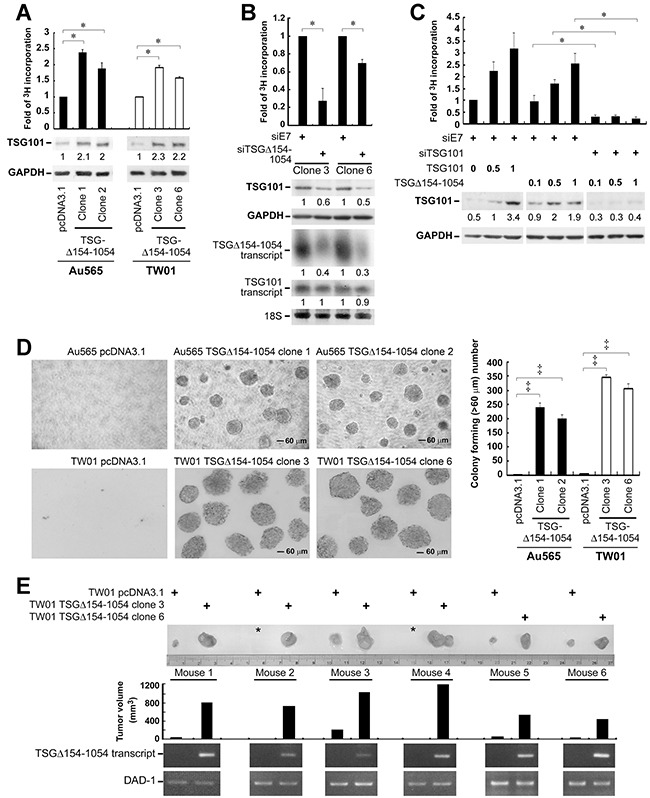
TSGΔ154-1054 augments the functional activities of TSG101 **A.** Proliferation of TSGΔ154-1054 stably expressed Au565 and TW01 cells. Cells were cultured in medium in the presence of [^3^H]-thymidine for 3 days. The incorporation of [^3^H]-thymidine into cells was counted. The protein amounts of TSG101 and internal control GAPDH are indicated by parallel blotting of the whole-cell lysate. The intensities of TSG101 proteins are specified at the bottom of TSG101 blot after normalizing with their corresponding GAPDH densities. **B.** Proliferation of TSGΔ154-1054 stable lines upon TSGΔ154-1054 depletion. siTSGΔ154-1054 and siE7 (an irrelevant siRNA control) were transfected twice into TW01 TSGΔ154-1054 lines. The transfectants were then reseeded onto 6-well plate for [^3^H]-thymidine incorporation assay (top), as well as western blot (middle) and northern blot (bottom) analysis. Level of 18S serves as loading control. The intensities of TSG101 proteins and TSG101/TSGΔ154-1054 transcripts are shown after standardizing with GAPDH and 18S, respectively. **C.** Effect of TSGΔ154-1054 on TSG101-induced cell proliferation. TW01 cells transfected with siE7 and siTSG101 for two passages were reseeded and transfected again with the respective plasmids as indicated. [^3^H]-thymidine incorporation assay was carried out and the expression of TSG101 was assessed by western blotting (Lower). Above each individual graph (A-C) is a representative result from three independent experiments performed in duplicate (mean ± SD, *p < 0.05 by two-tailed t test). **D.** Effect of TSGΔ154-1054 on anchorage-independent cell growth. Soft agar colony formation assay was achieved, and the forming foci numbers of Au565 and TW01 stable lines were counted at day 30 and day 14, respectively. Magnification is 100x. Above each individual graph is a representative result from three independent experiments performed in triplicate (mean ± SD, ^‡^*p* < 0.005 by two-tailed t test). **E.** Typical tumor growth of TW01 TSGΔ154-1054 stable lines in nude mice. The photograph demonstrated a comparable size difference of control vector- and TSGΔ154-1054 stable clone-derived tumors. Asterisk denotes tumor mass is undetectable. The expression of TSGΔ154-1054 transcript in tumor mass was assessed by RT-PCR (bottom). This animal experiment was repeated twice (n=20), and the TSGΔ154-1054-expressing xenografts of these mice (60%, 12/20) developed into large tumors in 3 to 4 weeks after initial injection (*p*<0.005, two-tailed t-test). A representative result is shown.

Given the demonstrated role for TSG101 in promoting cell proliferation [7], we postulated that this activity of TSG101 may be increased as a result of TSGΔ154-1054 stabilizing TSG101. In a [^3^H]-thymidine incorporation assay assessing cell proliferation, Au565 and TW01 cells stably expressing TSGΔ154-1054 showed a tendency to proliferate rapidly (Figure 5A). A reduced proliferation rate of TW01 TSGΔ154-1054 stable lines and a concurrent diminution in TSG101 protein level were shown when TSGΔ154-1054 was knocked down by its specific targeting siTSGΔ154-1054 (Figure 5B). Remarkably, when deprived of TSG101 by siTSG101, TSGΔ154-1054 could no longer account for the proliferative effect on TW01 cells (Figure 5C). These data imply the enhancement of cell proliferation by TSGΔ154-1054 is a consequence of TSGΔ154-1054 stabilizing TSG101.

Because TSGΔ154-1054 stabilizes TSG101, it is suggested that the oncogenicity of TSG101 is increased in the presence of TSGΔ154-1054. Impressively, the clonal proliferation of TSGΔ154-1054 stable lines in soft agar culture was enhanced (Figure 5D), in accord with the ability to promote cell proliferation (Figure 5A). Moreover TSGΔ154-1054 stably expressing cells increased *in vivo* tumor formation in nude mice (Figure 5E).

### TSGΔ154-1054 splice variant preferentially expressed in p53-mutated breast cancer specimens

The mechanism of TSGΔ154-1054 formation is still unclear [19]. We detected p53 mutation by polymerase chain reaction (PCR)-based amplification of each exon of p53 and the resulting amplicons were subjected to sequence analysis. The mutation was confirmed by a comparison to the nucleotide sequence of wild-type p53. TSGΔ154-1054 is preferentially expressed in p53-mutated breast cancer specimens as shown in Table 1 (Chi-square test; *p*=0.0001) and Supplementary Figure S3A, which reveals that p53 is a critical factor that prohibits the occurrence of TSG101 aberrant splicing. To clarify the importance of intact p53 in obstructing the expression of TSGΔ154-1054, we knocked down endogenous p53 in TW01 cells with p53 specific siRNA (sip53), and overexpressed wild-type p53 in p53-null Saos-2 cells. As a result, TSGΔ154-1054 was transcribed upon the depletion of p53 in TW01 cells; likewise, TSGΔ154-1054 was present in the p53-null Saos-2 cells, but lost immediately after p53 was ectopically expressed (Supplementary Figure S3B). This explains that the expression of TSGΔ154-1054 is prominently inhibited in cells with normal p53 activity.

**Table 1:** Correlation of p53 mutation and TSGΔ154-1054 expression in breast cancer

	p53 wild-type	p53 mutation^*^	*p* value^‡^
**TSGΔ154-1054 (−)**	60 (77.9 %)^†^	12 (37.5 %)	0.0001
**TSGΔ154-1054 (+)**	17 (22.1 %)	20 (62.5 %)	
Total case numbers	77	32	

* p53 mutation was detected by polymerase chain reaction (PCR)-based amplification of each exon of p53 and the resulting amplicons were subjected to sequencing analysis. The mutation was confirmed by a comparison to the nucleotide sequence of wild type p53.

† Number of breast cancer biopsies harbored wild-type p53 and without TSGΔ154-1054 expression detected by RT-nested PCR (percentage of case number from total 77 p53 wild-type population).

‡ Chi-square test was used to analyze the correlation between TSGΔ154-1054 positivity and the presence of p53 mutation.

## DISCUSSION

TSGΔ154-1054 is predominantly expressed in tumor tissues [14, 15, 17, 18] and the presence of TSGΔ154-1054 correlates with neoplastic progression [15]. We have found a direct association between TSGΔ154-1054 expression and increased TSG101 protein in both NPC and breast cancer patients. These findings suggest aberrant interplay between TSG101 and its splicing variant TSGΔ154-1054 in cancerous tissues. Furthermore, in unpublished observations TSGΔ154-1054 enhances the endogenous and exogenous protein levels of TSG101 in NPC TW01 cell lines. Although the biological significance of TSGΔ154-1054 has already been suggested by its association with tumor progression [15, 19], the mechanism of its role was unknown. In the present study, we are the first to demonstrate that TSGΔ154-1054 prolongs the half-life of TSG101 protein which further consolidates the hypothesis mentioned above.

TSGΔ154-1054 stabilizes TSG101 by interfering with the Ub-proteasome-dependent degradation of TSG101 conferred by Tal which was demonstrated in Figure 3. The ability of TSGΔ154-1054 to reduce TSG101 ubiquitination seems attributable to its binding Tal, minimizing Ub transmission from Tal to TSG101. TSGΔ154-1054 is distinct from other splice variants in regulating its native protein stability, as exemplified by p47, a splice variant of p53. By way of direct interaction with p53 to form p53/p47 heterodimers, p47 enhances p53 nuclear export and thereby protects p53 from MDM2-mediated polyubiquitination [29]. TSGΔ154-1054 does not directly interact with TSG101 but competes with TSG101 for binding Tal and subsequently inhibits the polyubiquitination of TSG101 which is evident in Figure 4. The consequence of Tal-TSGΔ154-1054 interaction may diverge from that of Tal-TSG101, wherein Tal binds and subsequently degrades TSG101, yet Tal may not exert its proteolytic activity on TSGΔ154-1054 even if Tal binds TSGΔ154-1054. This is deduced by the fact that the lysine residues in C-terminal S-box of TSG101, which are accessible to polyubiquitination transferred by Tal [25], are absent in TSGΔ154-1054 because of premature translational termination, thereby causing the failure of action of Tal ligase on TSGΔ154-1054. Another mechanism stabilizing TSG101 has been reported; VPS28 binds and masks the C-terminal S-box of TSG101 for preventing Tal-targeted proteolysis, leading to stabilization of TSG101 and promoting ESCRT function [25]. In our observation, inhibition of Tal due to the overexpression of TSGΔ154-1054 in cancer cells may add to the effect of VPS28 to effectively prevent TSG101 degradation.

It has been known that TSG101 critically controls cell proliferation in both mouse embryonic tissues and human cancer cells [5–7]. Centrosome protein 55 (Cep55), CBP/p300-interacting transactivator with ED-rich tail 2 (CITED2), hypoxia-inducible factor 1α (HIF-1α), and the MAPK/ERK signaling pathway, have been demonstrated to participate in TSG101-mediated proliferation [21, 30, 31]. Stabilization of TSG101 by TSGΔ154-1054 may act synergistically with these factors to increase the proliferative activity of TSG101. Coexistence of TSGΔ154-1054 and TSG101, most prominently, accounts for the tumor cell aggressiveness including increasing capacity for colony formation, as well as enhancing tumor xenograft growth in nude mice, in addition to increased proliferation. Collectively, TSGΔ154-1054 stabilizing TSG101 prompts the development of tumor malignancy.

TSGΔ154-1054 formation may be due mainly to re-splicing of the mature (spliced) TSG101 mRNA [32]. Besides, it has been proven that absence of p53 frequently leads to increased activity of SRSF6 protein (formerly SRp55), a member of the serine-arginine-rich (SR) protein family, which are essential for splicing regulation [33]. In accord with this, the accumulation of the TSGΔ154-1054 splice variant is preferentially expressed in p53-mutated and inactivated cancer cell lines [34] and in the p53-depleted TW01 cells, as well as the p53-mutated breast cancer specimens from our own patient group. Moreover, activation of p53 by gamma-irradiation diminishes the variant expression [34]. All of these findings indicate that p53 is a critical factor that prohibits TSG101 aberrant splicing, probably through constraining the activities of SR proteins.

The TSGΔ154-1054 protein with predicted molecular weight of 3 kDa had an apparent size of ~18 kDa in immunoblotting of NPC cells and tissues as shown in Supplementary Figure S1. The prediction of the post-translational modification with NetGlycate 1.0 shows possible glycation at its K9 and K10 residues. It is now recognized that a number of small protein molecules undergoing post-translational modification are protected from degradation, which may in turn regulate bioactivity. One example is osteocalcin, a relatively small protein of 5.7 kDa which is γ-carboxylated to yield a ~12 kDa protein [35]. Glycation can occur in cellular proteins through binding of glucose to the amino groups of proteins. This may lead to aberrant cell signaling, loss of genetic fidelity, and pathogenesis of human cancer [36].

Taken together, we present a novel role of TSGΔ154-1054 splicing variant, stabilizing TSG101 protein during tumorigenesis. The direct competition between TSG101 and TSGΔ154-1054 for interaction with Tal demonstrates a novel mechanism for posttranslational protein regulation; and, by this means TSGΔ154-1054 is implicated in the TSG101-associated oncogenic events and therefore, be a potential target for cancer therapy.

## MATERIALS AND METHODS

### Primary tumor specimens and cell lines

109 breast cancer primary tumor specimens were provided by the Departments of Surgery and Pathology of National Taiwan University Hospital. All procedures were in accordance with the ethical standards of the committee on human experimentation of College of Medicine, National Taiwan University. TW01 [37], TW04 [37] and HONE1 [38] were the human NPC cell lines. TSGΔ154-1054 stably expressed in TW01 and Au565 (breast cancer cell line) were established in our laboratory. U2OS, a human osteosarcoma cell line which harbors a single copy of *MDM2* gene and which expresses a negligible amount of MDM2 was also used. Besides, Saos-2, a p53-null human osteosarcoma cell line, was used in this study. Au565, U2OS, and Saos-2 cell lines were purchased from ATCC. All cell lines were grown in Dulbecco's modified Eagle's medium (DMEM; Hyclone) supplemented with 10% fetal calf serum, L-glutamine, and penicillin-streptomycin. The TSGΔ154-1054 and its paired control stable lines were maintained in G418-selective DMEM.

### Plasmid construction and transfection

Myc-Ub expression plasmids were gifts from Dr. Zee-Fen Chang (Institute of Biochemistry and Molecular Biology, College of Medicine, National Taiwan University, Taipei, Taiwan). pcDNA3.1-HA-Tal was a present from Dr. Yosef Yarden (Department of Biological Regulation, The Weizmann Institute of Science, Rehovot, Israel). pCMV-HA-MDM2 plasmid was kindly provided by Dr. Tzu-Hao Cheng (Institute of Biochemistry and Molecular Biology, National Yang-Ming University, Taipei, Taiwan). pcDNA-wild-type p53 was a gift from Dr. Sheau-Yann Shieh (Institute of Biomedical Sciences, Academia Sinica, Taipei, Taiwan). Constructs bearing TSG101 and TSGΔ154-1054 were amplified by PCR and cloned into pcDNA3.1 (Invitrogen, Carlsbad, CA), and pGEX-4T-1 (Novagen, Madison, WI) to yield GST-fusion protein. To generate the GST-HA-Tal expression plasmid, we cloned the HA-Tal plasmid backbone from pcDNA3.1 into pGEX-4T-1. A PCR-based site-directed mutagenesis technique using QuikChange Lightning Site-Directed Mutagenesis kit (Agilent Technologies) was adapted to construct the pGEX-4T-1-based HA-Tal deletion mutants. The primer design and PCR conditions were optimized and set specifically according to the manufacturer's instructions. The details of the construction of pSuper-siTSG101, -sip53 and -siE7 (targeting human Papillomavirus type 18 oncoprotein E7) have been described in our previous publication [20, 39]. pSuper vector carrying the siTSGΔ154-1054-expressing cassette was created by cloning a short overlapping oligonucleotide siRNA template (5′-GACCTAACTCTCCCTTATA-3′), which is complementary to the TSG101 sequence nucleotides 145 to 153 (GACCTAACT) that joined to nucleotides 1055 to 1064 (CTCCCTTATA).

Plasmid DNA transfection was achieved by using TransFast™ Transfection reagent (Promega), according to the manufacturer's instructions.

### Western blot

Total proteins were extracted by lysis buffer containing 3% SDS, 2 M urea, and 2% 2-mercaptoethanol. The protein lysates were then resolved by electrophoresis on a 10% SDS-polyacrylamide gel and transferred onto a Hybond-C membrane (Amersham Biosciences). After blocking in 4% skim milk-containing washing buffer (100 mM Tris-HCl [pH 7.4], 150 mM NaCl, 0.2% Tween 20) at room temperature for 1 h, the blots were incubated overnight with primary antibodies directed against TSG101 (GeneTex), HA (Babco), Myc (a gift from Dr. Zee-Fen Chang, Institute of Biochemistry and Molecular Biology, College of Medicine, National Taiwan University), Ub (Santa Cruz Biotechnology), p53 (Santa Cruz Biotechnology), homemade Glutathione-S-transferase (GST) and GAPDH (Biodesign), at 4°C. Three rounds of 10-min washes with washing buffer were performed on the antibody-bound blots, prior to the l h incubation of horseradish peroxidase-conjugated secondary antibodies at room temperature. Eventually, the detected proteins were revealed by enhanced chemiluminescence (PerkinElmer Life Sciences) and exposure on X-ray films. To specifically detect the endogenous TSGD154-1054 protein, NPC cell lines and tissues were lysed by RIPA lysis buffer supplied with protease inhibitor mixture (Roche) and 10 μg/mL p-amidinophenyl methane sulphonyl fluoride hydrochloride. Total protein lysates (20 μg) were resolved on 10-20% Mini-PROTEAN® Tris-Tricine gel (Bio-Rad), and transferred onto PVDF blotting membranes with 0.2 μm pore size (Amersham Biosciences) at 250-mA constant current for 30 min. The membranes were blocked with 4% skim milk-containing washing buffer for 1 h at room temperature, and subsequently probed with primary anti-TSG101 amino terminal end antibody (ab50582, Abcam) at 4°C overnight.

### RNA isolation and RT-PCR

Total RNAs were purified by TRIzol reagent (Invitrogen). Complementary DNAs were generated from 1 μg of DNase I (Gibco BRL)-treated total RNAs with random hexamers (Gibco BRL) and SuperScript™II RNase H^−^ reverse transcriptase (Gibco BRL) according to the outline of manufacturer's instructions. For the nested-PCR of TSG101, first round PCR amplification was carried out in a 10 μL final volume of 1 μL cDNA, 1x PCR reaction buffer, 0.2 μM dNTPs, 0.1 U AmpliTaq Gold (Applied Biosystems), 2 μM each of P1 (5′-CGGTGTCGGAGAGCCAGCTCAAGAAA-3′) and P2 (5′-CCTCCAGCTGGTATCAGAGAAGTCAGT-3′) primers. The amplification was started with denaturing cDNA at 95°C for 5 min, then 25 cycles of 95°C for 50 sec, 65°C for 30 sec, and 72°C for 1 min. The final PCR products were diluted with ddH_2_O (1:4) and 0.5 μL of these diluents served as templates for the second round PCR, using P3 (5′-AGCCAGCTCAAGAAAATGGTGTCCAAG-3′) and P4 (5′-TCACTGAGACCGGCAGTCTTTCTTGCTT-3′) nested primers. The second round of PCR amplification was performed with 30 cycles of 95°C for 50 sec, 67°C for 30 sec, and 72°C for 1 min. An agarose gel (1.5 %) containing 0.1 % ethidium bromide was utilized to analyze the PCR products and the results were photographed. For cells transfected with TSGΔ154-1054, the TSGΔ154-1054 cDNA was detected using PCR primers P3 and P4, and amplified under the corresponding PCR condition.

The detection of Defender Against Death-1 (DAD-1), an internal control, was optimized in a 10 ml final volume of PCR reaction containing 2 μL of cDNA, 1x PCR reaction buffer, 0.4 μM dNTPs, 1 U of ProZyme (Protech), and 0.4mM each of forward primer (5′-GCAGTTATGTCGGCGTCGGTAG-3′) and reverse primer (5′-GTTCTGTGGGTTGATCTGTATTC-3′). The amplification was conducted for 25 cycles of 94°C for 15 sec, 65°C for 15 sec, and 72°C for 30 sec.

### Northern blot

The denatured total RNA were electrophoresed on 2 % agarose gel and subsequently transferred onto an Immobilon-NY+ membrane (Millipore). Then, the UV-cross-linked RNA blots were pre-hybridized in ultrasensitive hybridization buffer (ULTRAhyb™, Ambion) for 2 h. The ^32^P-labeled DNA probes were generated by enzyme cutting the specific DNA fragment from the TSGΔ154-1054 and TSG101 expression plasmids. These probes were hybridized overnight to the blot in ULTRAhyb™ buffer at 42°C. Thereafter, the blots were washed stringently in 0.1 % SDS-containing 2x saline sodium citrate buffer, at 42°C, and the results were visualized by X-ray film exposure.

### Pulse-chase assay

Eighteen hours after transfection, the transfected cells were washed twice with PBS, and pulse labeled for 2 h with 3 μCi/ml of ^35^S-[Met] in a methionine-free labeling medium (DME/HIGH MODIFIED DMEM, JRH Biosciences). The remaining ^35^S-[Met] was chased by washing twice with cooled PBS and three times with DMEM (HyClone). Cells were fed subsequently with complete DMEM and harvested at the times indicated. Cell lysates were prepared using RIPA lysis buffer [50 mM Tris pH 8.0, 150 mM NaCl, 2 mM EDTA, 1% NP40, 0.1% SDS, protease inhibitor mixture (Roche)].

### CHX chase assay

Cells seeded onto 6-well plates were treated with cycloheximide (120 μg/mL) 18 hours post-transfection. Cells were then cultured for the time indicated and harvested for immunoblotting.

### Immunoprecipitation

Protein extracts were precleared with 50 % protein A sepharose (Amersham Biosciences), and incubated with 2 ug of anti-TSG101 (Novus) or -GFP (Millipore) antibodies overnight at 4°C. Thereafter, 100 μl of 50% protein A sepharose were added and incubated for an additional 2 h. The sepharose bead-bound immunocomplexes were washed three times with 1x PBS and dissolved in 2x SDS sample buffer.

### *In vitro* binding assay and *in vitro* competitive binding assay

The *in vitro* translated proteins were prepared using TNT T7 Quick Coupled Transcription/Translation System (Promega). GST-TSG101 and GST-TSGΔ154-1054 proteins were synthesized in *E.coli* (BL21-DE3 strain) and purified by absorption to glutathione-Sepharose (Sigma). These proteins were then incubated in buffer A (1% Triton X-100, 20 mM Tris-HCl, 140 mM NaCl, 1 mM EGTA, 10% (v/v) glycerol, 1.5 mM MgCl_2_, 1 mM DTT, 1 mM sodium vanadate, 50 mM NaF, 1 μg/mL aprotinin, 10 μg/mL p-amidinophenyl methane sulphonyl fluoride hydrochloride, pH 8.0) for 2 h at 4°C. Complexes that adhered to bead-bound proteins were washed thoroughly with buffer B (20 mM Tris-HCl, 150 mM NaCl, 1% NP-40, pH 8.0), and analyzed by western blotting. For *in vitro* competitive binding assay, the *in vitro* translated HA-Tal and TSGΔ154-1054 (the competitor) were mixed with glutathione-Sepharose-immobilized GST-TSG101 in buffer A, in a total volume of 100 μL. The binding mixtures were incubated at 4°C for 2 h. Unbound proteins were removed by a subsequent washing with buffer B.

### *In vitro* ubiquitination assay

The GST-pulled down GST-HA-Tal was treated with thrombin (Amersham Pharmacia Biotech) at 25°C for 16 h to remove the conjugated GST. Next, 80 μL ubiquitination reaction buffer (25 mM Tris-HCl, pH 7.6, 100 mM NaCl, 5 mM MgCl_2_, 2 mM ATP, 1 mM DTT), containing 150 ng E1 (Biomol International), 150 ng E2 (BostonBiochem), 300 ng HA-Tal, 5 μg Ub (Sigma), 150 μg glutathione-Sepharose-immobilized GST-TSG101, and 24 μL *in vitro* translated TSGΔ154-1054, were prepared. The reaction was incubated at 37°C for 90 min and terminated by four washes in buffer containing 1% Triton and 1% SDS.

### [^3^H]-thymidine incorporation assay for cell proliferation

1 × 10^4^ cells plated onto each well of 6-well plates were incubated with 3 μCi/well of [methyl-^3^H]-thymidine (PerkinElmer Life Sciences) for 72 h. After washing 3 times with cold 1x PBS, cells were dissolved in 0.1 M NaOH. Incorporation of [^3^H]-thymidine was measured with a liquid scintillation counter (LS6000TA, Beckman Coulter).

### Soft agar colony formation assay

1 × 10^4^ cells were suspended in complete DMEM containing 0.33% low-melting-temperature agarose and seeded onto 60-mm plates layered with solidified modified Eagle's medium-10% fetal calf serum-0.3% tryptose phosphate broth-0.5% agarose. Cells were cultured at 37°C for 2-4 weeks. A crystal violet stain was performed on the forming colonies.

### Tumor growth in nude mice

3 × 10^6^ of TSG101Δ154-1054 stably expressing TW01 cells and the control lines were resuspended in 200 μL PBS, and inoculated subcutaneously into the right and left flanks, respectively, of 5-week-old BALB/c athymic nude mice. All these mice were maintained in accordance with procedures of the Institutional Animal Care and Use Committee of National Taiwan University. Mice with developed tumors were sacrificed at 50 days after initial injection. The tumor masses were surgically removed, measured, and subjected to RNA extraction.

### Statistical analysis

Statistical programs in Microsoft Excel (Microsoft Co.) were utilized for data analysis. Student's t-test was used to test for significant differences in continuous variables between groups. A *p*-value below 0.05 was considered statistically significant.

## SUPPLEMENTARY FIGURES


