# Supplementary material for: TSGΔ154-1054 splice variant increases TSG101 oncogenicity by inhibiting its E3-ligase-mediated proteasomal degradation
Source: Oncotarget. 2016 Jan 22;7(7):8240–52. doi: 10.18632/oncotarget.6973 (PMC4884989; doi:10.18632/oncotarget.6973)
Supplement: Supplementary file 1 [file oncotarget-07-8240-s001.pdf]

SUPPLEMENTARY FIGURES

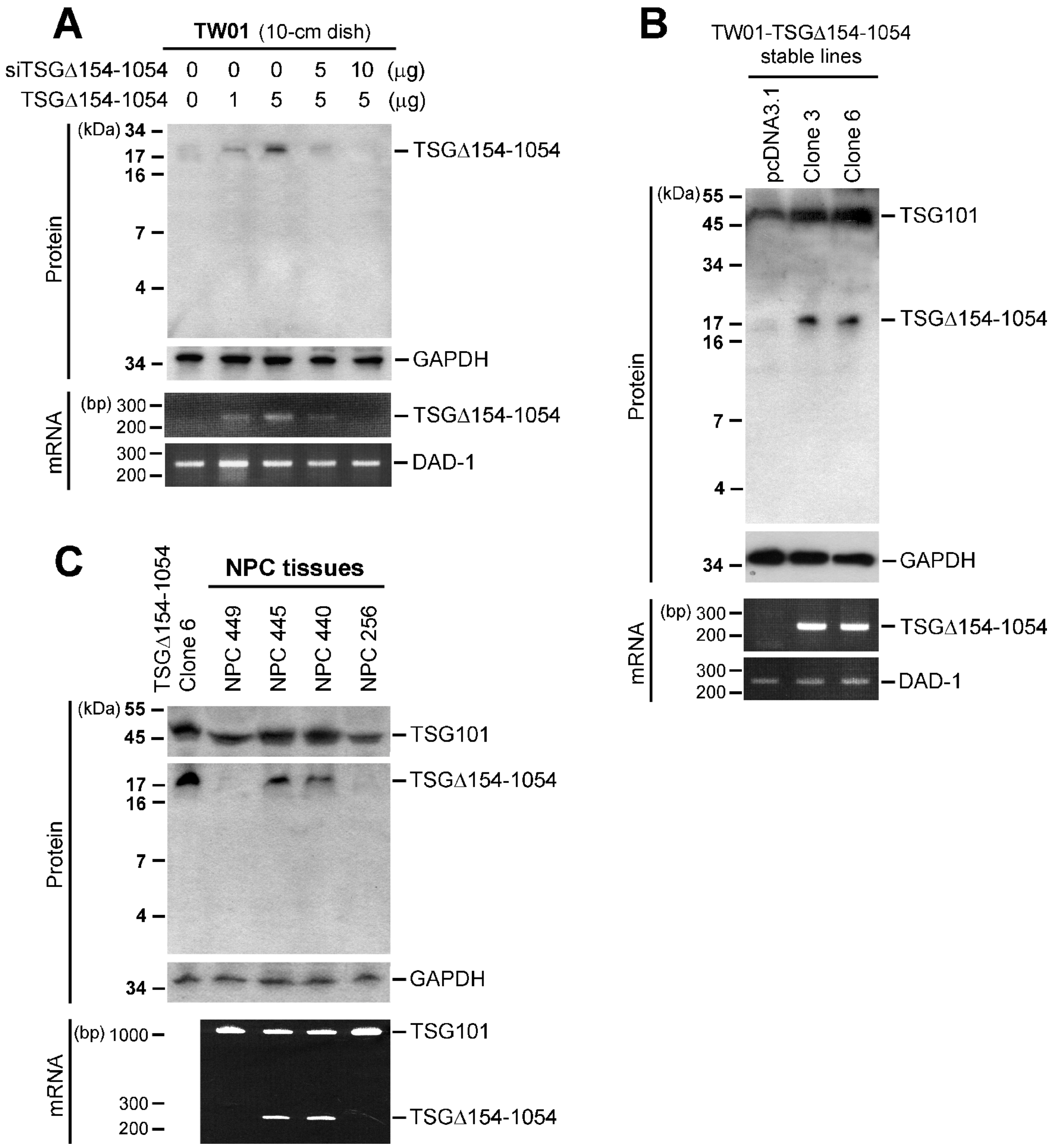

Supplementary Figure S1: Expression of TSGΔ154-1054 protein. A-B. Total protein lysates harvested from TW01 cells transfected with plasmids indicated (A) or stably expressed TSGΔ154-1054 (B) were resolved in 10–20% Mini-PROTEAN® Tris-Tricine gels, and transferred onto 0.2 μm PVDF membranes. Immunoblotting of TSGΔ154-1054 protein was performed using anti-TSG101 antibody-aminoterminal end (Upper). Detection of GAPDH serves as an internal control. In parallel, total RNAs were isolated from these cells and subjected to TSGΔ154-1054-specific RT-PCR analysis using primers P3 and P4 (Lower). RT-PCR analysis of DAD-1 expression was used as a cDNA loading control. **C.** Total proteins and RNAs were extracted from primary NPC tissues, and applied for western blot (Upper) and RT-nested PCR (Lower) assays, respectively. The protein levels of TSG101 and TSGΔ154-1054 were detected using anti-TSG101 antibody-aminoterminal end. TSGΔ154-1054 clone 6 stable line was included as a positive control for TSGΔ154-1054 expression.

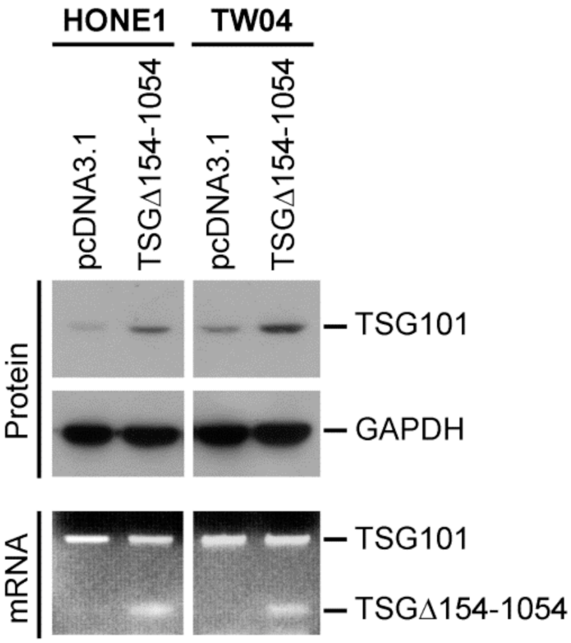

Supplementary Figure S2: Overexpression of TSGΔ154-1054 increases the endogenous TSG101 protein in NPC cell lines, HONE1 and TW04. Plasmid expressing TSGΔ154-1054 and control vector were transfected into HONE1 and TW04 cells. These cells were harvested for western blot (Upper) and TSG101-specific RT-nested PCR assays at 24 h post-transfection.

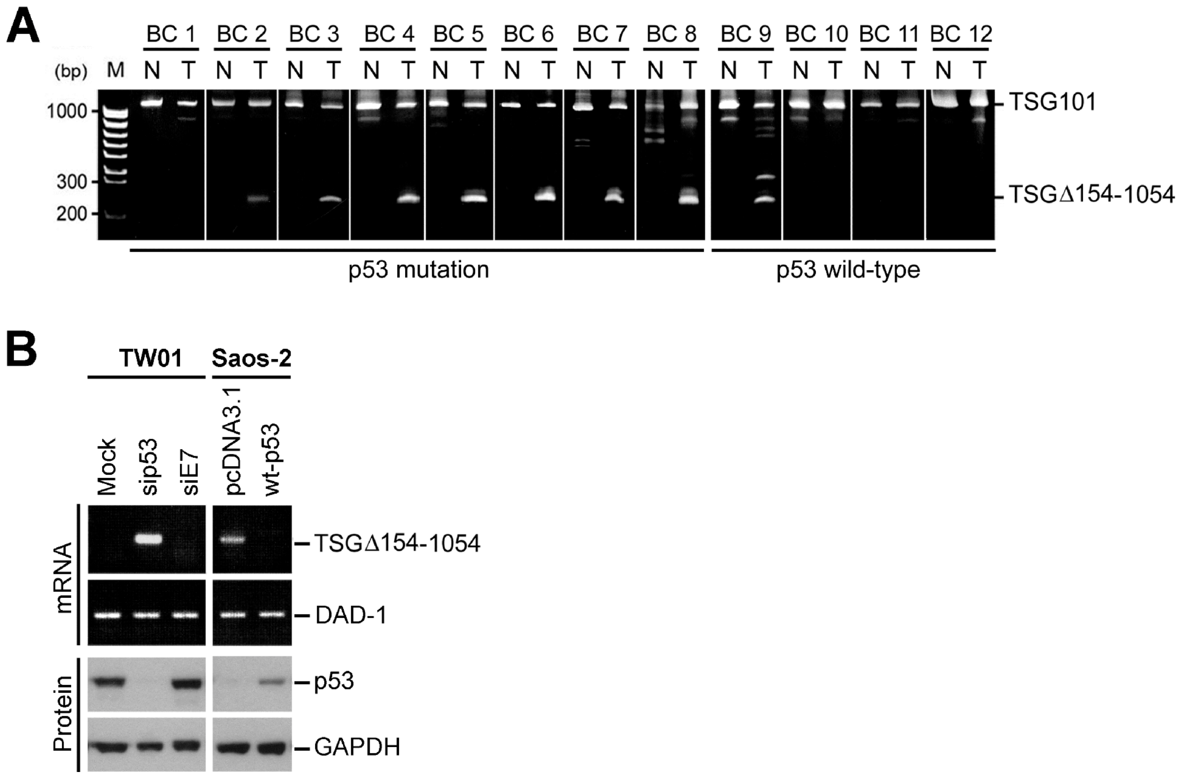

Supplementary Figure S3: An intact p53 prevents the expression of TSGΔ154-1054. A. RT-nested-PCR of TSG101 splicing products in tumor specimens (T) and their normal counterparts (N) of breast cancer (BC). BC tissues that harbored wild-type or mutated p53 were indicated. **B.** TW01 and Saos-2 cells transfected with plasmids indicated were subjected to TSGΔ154-1054-specific RT-PCR (Upper) and western blot analysis. DAD-1 and GAPDH serve as internal controls for total mRNA and protein levels, respectively.
